# Supplementary material for: Covalent TCR-peptide-MHC interactions induce T cell activation and redirect T cell fate in the thymus
Source: Nat Commun. 2022 Aug 23;13:4951. doi: 10.1038/s41467-022-32692-4 (PMC9399087; doi:10.1038/s41467-022-32692-4)
Supplement: Supplementary file 1 — Supplementary Information [file 41467_2022_32692_MOESM1_ESM.pdf]

SUPPLEMENTARY INFORMATION

Covalent TCR-peptide-MHC interactions induce T cell activation and redirect T cell fate in the thymus

Christopher Szeto[1,2], Pirooz Zareie[1], Rushika C. Wirasinha[1], Justin B. Zhang[1], Andrea T. Nguyen[1,2], Alan Riboldi-Tunnicliffe[3], Nicole L. La Gruta[1], Stephanie Gras[1,2] & Stephen R. Daley[1,4]*

[1] Infection and Immunity Program and Department of Biochemistry and Molecular Biology, Biomedicine Discovery Institute, Monash University, Clayton 3800, Victoria, Australia.

[2] Department of Biochemistry and Genetics, La Trobe Institute for Molecular Science, La Trobe University, Bundoora 3086, Victoria, Australia.

[3] Australian Synchrotron, Australian Nuclear Science and Technology Organisation, Clayton 3800, Victoria, Australia.

[4] Centre for Immunology and Infection Control, School of Biomedical Sciences, Faculty of Health, Queensland University of Technology, Brisbane 4000, Queensland, Australia.

These authors contributed equally: Christopher Szeto, Pirooz Zareie.

These authors jointly supervised this work: Nicole L. La Gruta, Stephanie Gras, Stephen R. Daley

Correspondence and requests for materials should be addressed to N.L.L.G. (email: nicole.la.gruta@monash.edu) or to S.G. (S.Gras@latrobe.edu.au) or to S.R.D. (s5.daley@qut.edu.au).

Supplementary Information Contents:

- 9 Supplementary Figures
- 3 Supplementary Tables

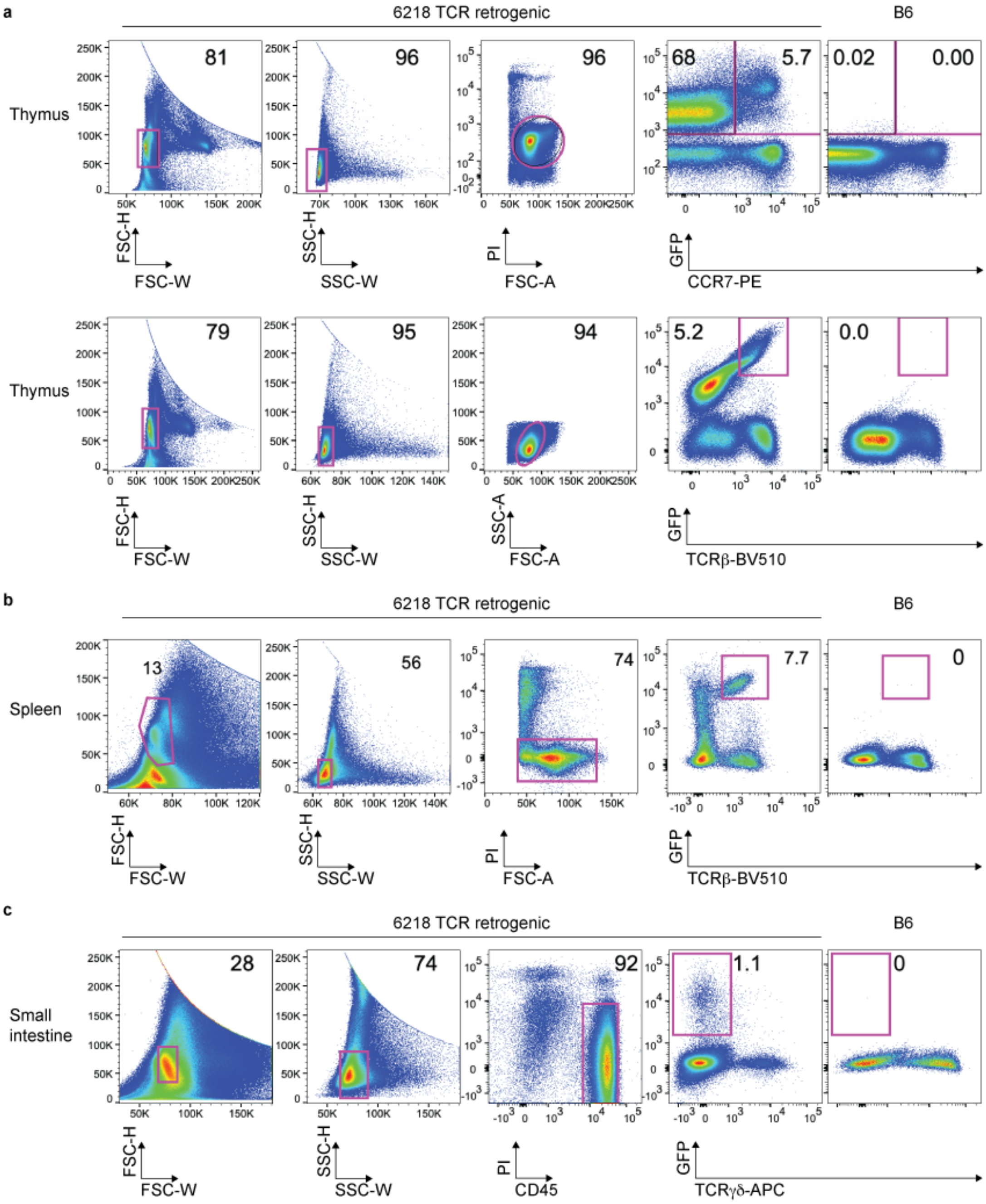

Supplementary Figure 1. **FACS gating strategy for analysis of TCR-retrogenic mice. a-c** Flow cytometric gating strategies show samples from the **a** thymus, **b** spleen, and **c** small intestine, of mice expressing the 6218 TCR made using *Rag1*$^{-/-}$ BM and recipients as represented in Figure 1. Similar gates were used to make Figure 3a and Supplementary Figure 2. In each experiment, samples from a B6 mouse were analysed in parallel to provide negative controls for the detection of GFP^{+} cells.

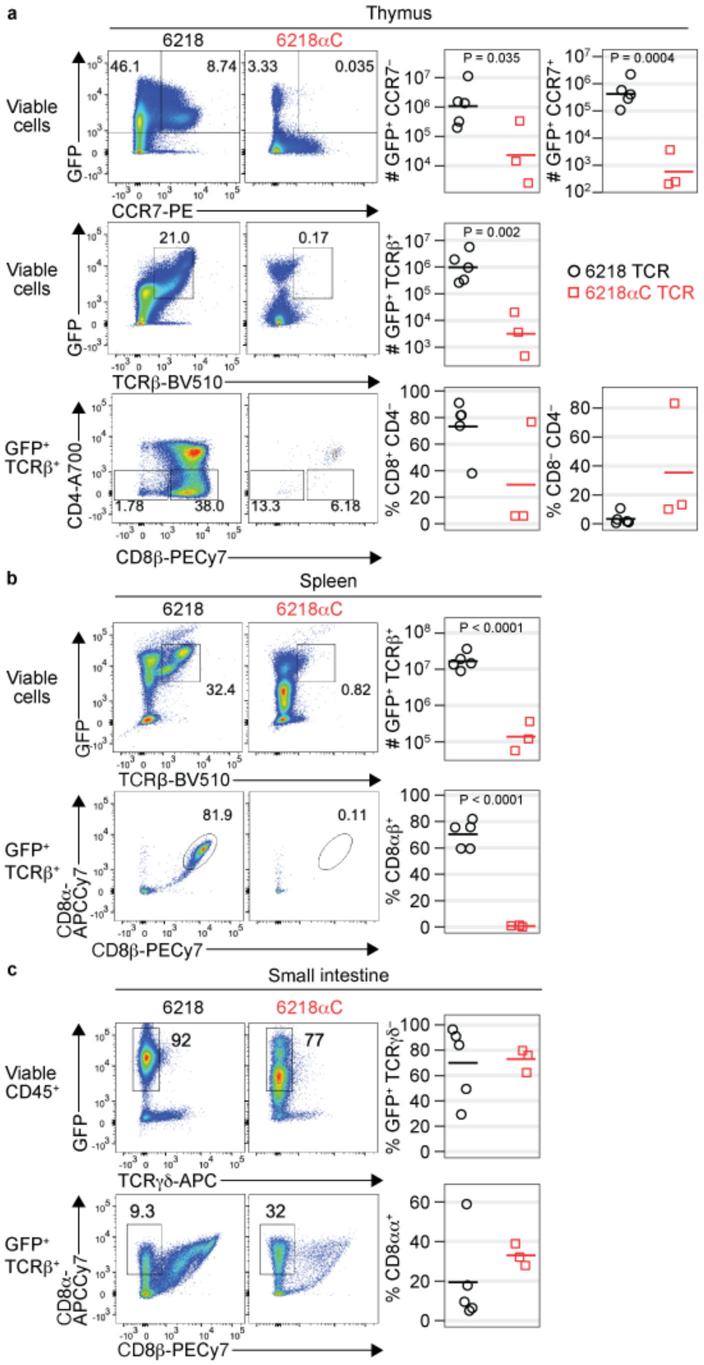

Supplementary Figure 2. **A CDR3 cysteine is sufficient to alter T cell fate.** BM cells pooled from *Tcra*$^{-/-}$ mice (5 female and 5 male, aged 169-207 d) were transduced with retroviruses encoding GFP and the 6218 or 6218αC TCR and then injected into irradiated female *Tcra*$^{-/-}$ mice (n = 5 for 6218; n = 3 for 6218αC). TCR-retrogenic mice were analysed 58 or 66 days after BM transfer at 123-149 days of age. **a** GFP/CCR7 (row 1) and GFP/TCRβ (row 2) phenotypes of thymocytes, with a gate for GFP^{+} TCRβ^{+} cells then analysed for CD4/CD8β (row 3). **b** GFP/TCRβ phenotype of splenocytes (top) with a gate for GFP^{+} TCRβ^{+} cells then analysed for CD8α/CD8β (bottom). **c** GFP/TCRγδ phenotype of small intestinal CD45^{+} cells with a gate for GFP^{+} TCRγδ^{-} cells then analysed for CD8α/CD8β (bottom row). Graphs show the absolute number (#) or the percentage of gated events as denoted on the y-axis. Each symbol in a graph represents 1 mouse and horizontal lines show the group mean; black for 6218 and red for 6218αC. P values were calculated using unpaired two-tailed t tests. Source data are provided as a Source Data file.

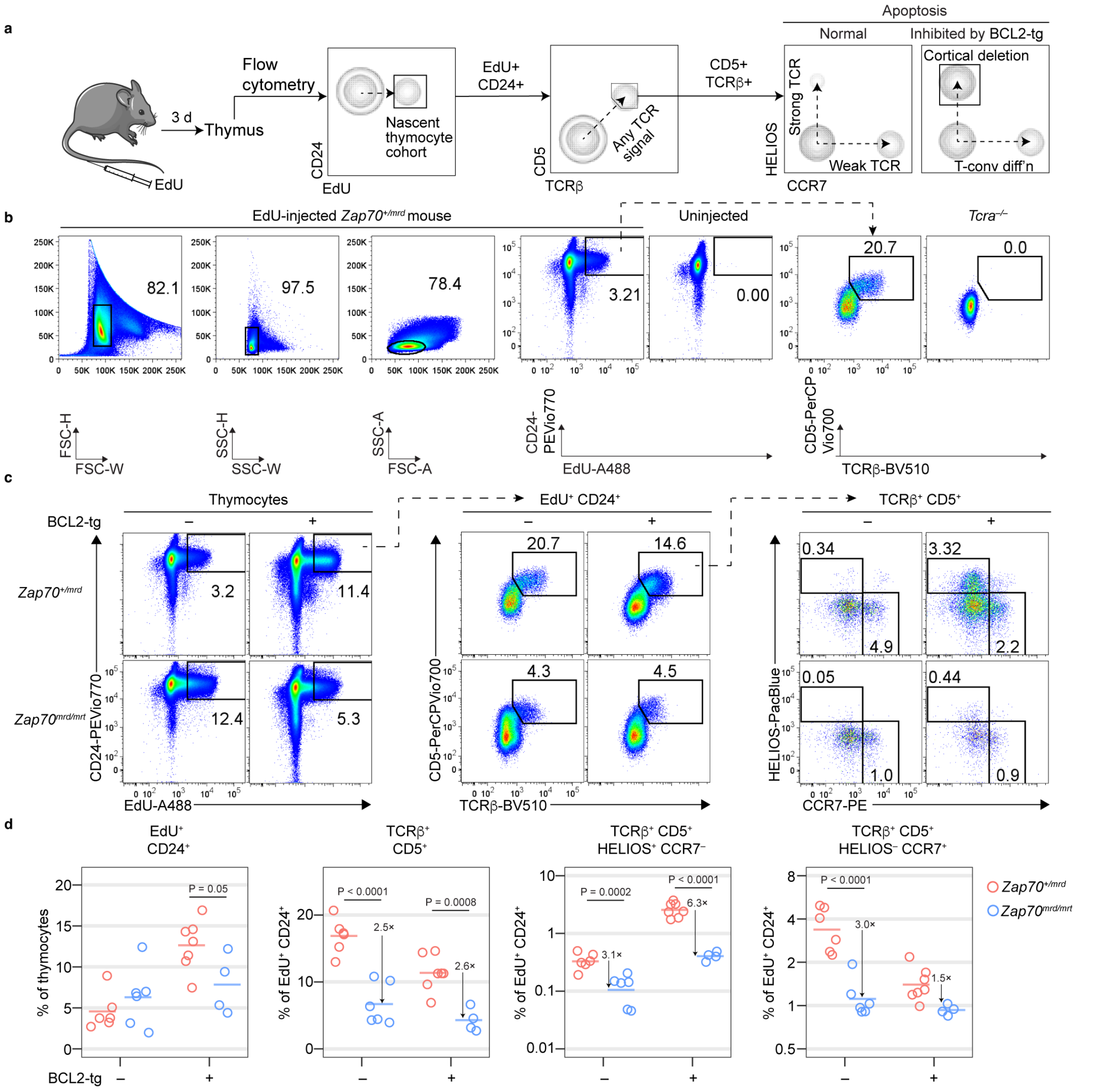

Supplementary Figure 3. **Disproportionate reduction of strongly TCR-signalled thymocytes in *Zap70*$^{mrd/mrt}$ mice. a** Quantifying deletion in CCR7^{-} thymocytes (cortical deletion). Three days after proliferating thymocytes incorporate 5-ethynyl-2'-deoxyuridine (EdU), nascent (EdU^{+} CD24^{+}) TCR-signalled (CD5^{+} TCRβ^{+}) cells were resolved into Helios^{+} CCR7^{-} and Helios^{-} CCR7^{+} subsets, which had received a strong or weak TCR signal, respectively. A human BCL2 transgene (*Tg*$^{(Vav\text{-}BCL2)1Jad}$, BCL2-tg)[61] inhibits apoptotic deletion, enabling the scale of cortical deletion to be measured. **b** FACS gates including uninjected and *Tcra*$^{-/-}$ negative controls. **c** Left panel shows CD24/EdU phenotype with a gate for EdU^{+} CD24^{+} (nascent) cells. Middle panel shows CD5/TCRβ phenotype of EdU^{+} CD24^{+} cells with a gate to identify CD5^{+} TCRβ^{+} (TCR-signalled) cells. Right panel shows Helios/CCR7 phenotypes of EdU^{+} CD24^{+} CD5^{+} TCRβ^{+} cells. **d** Graphs show frequencies of populations gated in **c** within the parent population stated on the y-axis; red for *Zap70*$^{+/mrd}$ (n = 4 female and 2 male BCL2-tg^{-}, 3 female and 4 male Bcl2-tg^{+}) and blue for *Zap70*$^{mrd/mrt}$ (n = 5 female and 1 male BCL2-tg^{-}, 2 female and 2 male Bcl2-tg^{+}). Horizontal lines show the group means compiled from 3 experiments. P values in **d** were determined using 2-way ANOVA with Sidak's multiple comparisons test. Source data are provided as a Source Data file.

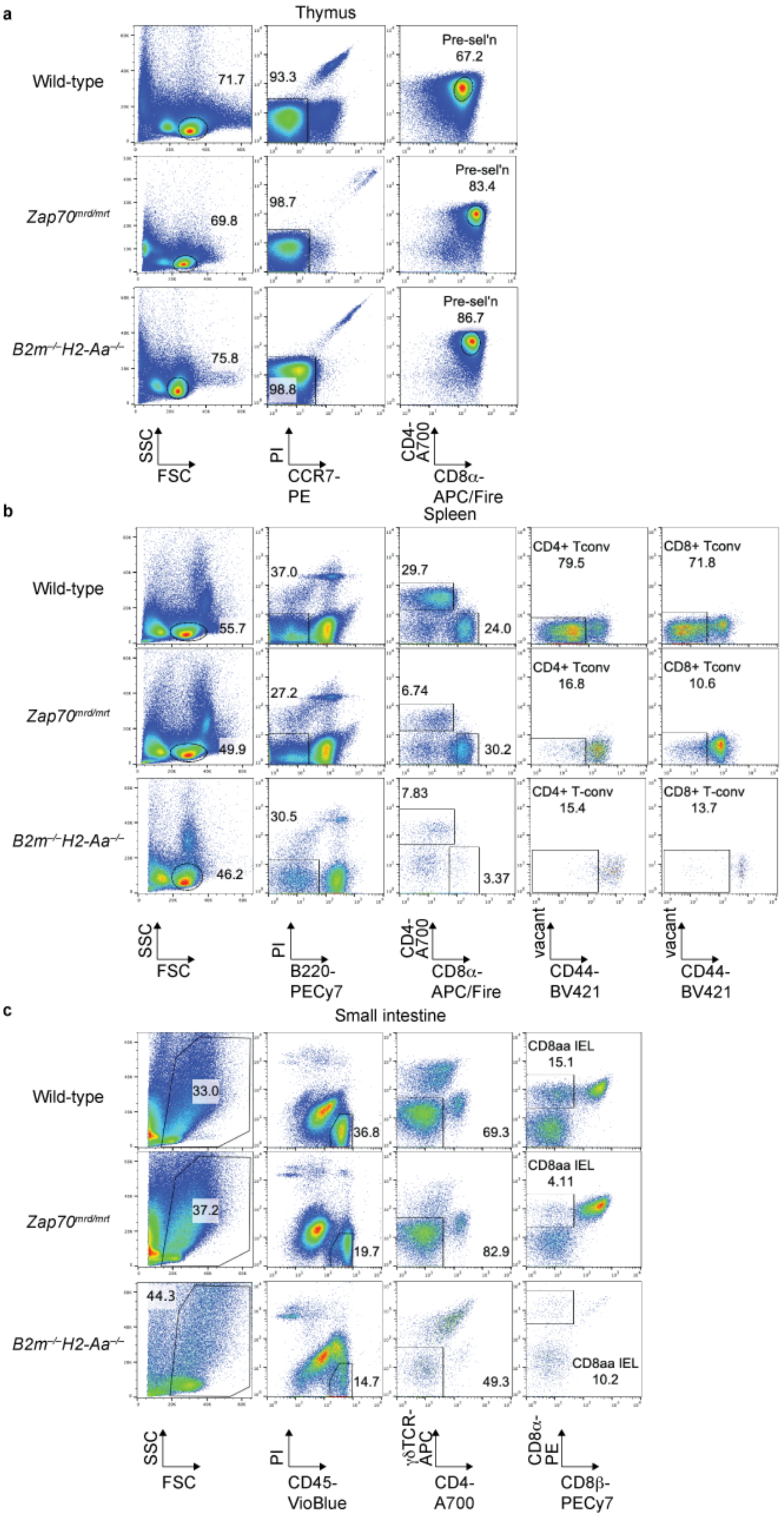

Supplementary Figure 4. **FACS sorting of polyclonal T cell subsets for TCR sequencing.** Plots show representative staining and gating used to sort the indicated T cell subsets from the **a** thymus, **b** spleen, or **c** small intestine, of wild-type, *Zap70*$^{mrd/mrt}$ or *B2m*$^{-/-}$*H2-Aa*$^{-/-}$ mice. Analysis of the TCR sequencing data is shown in Figure 3 b-d.

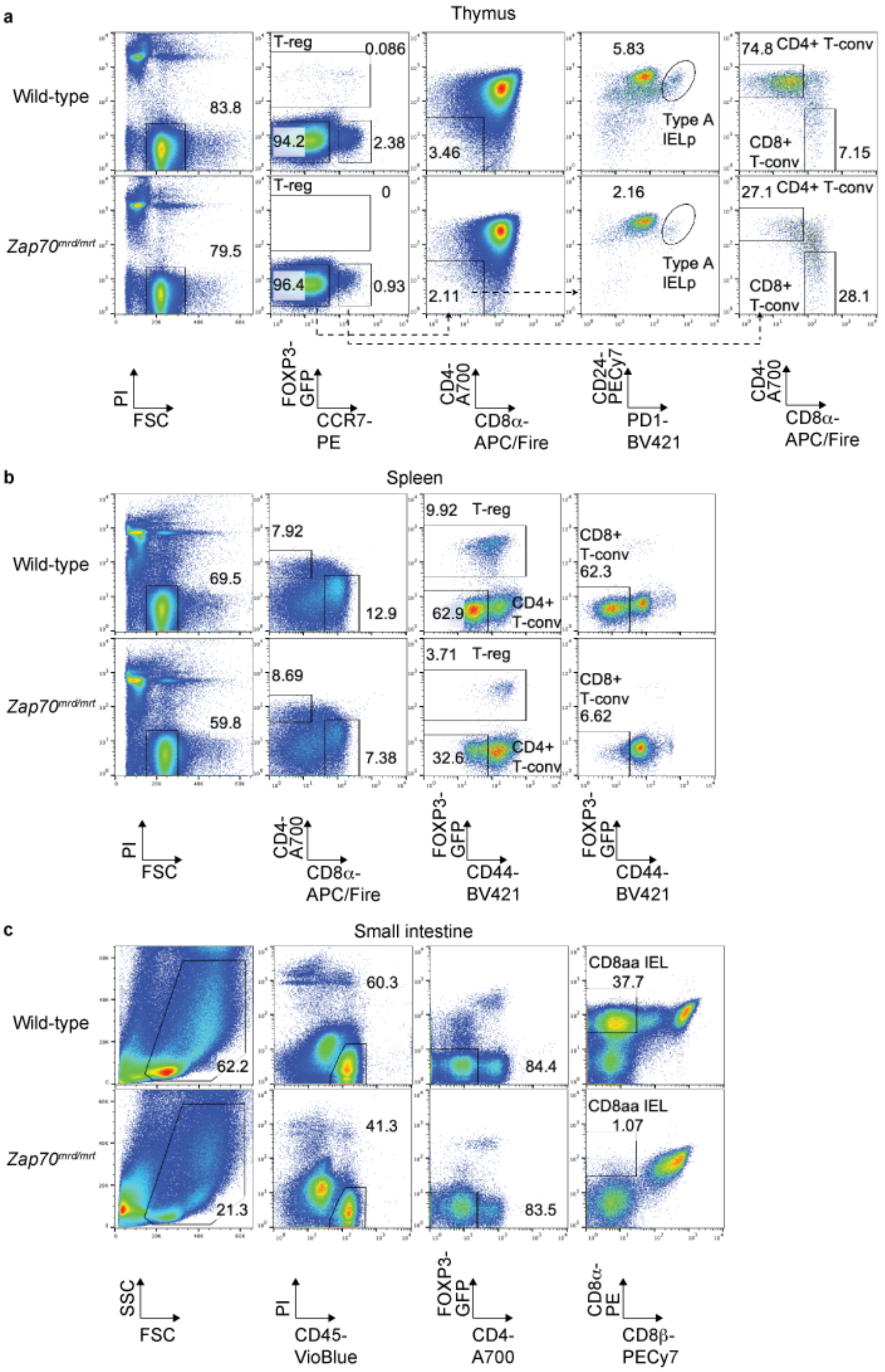

Supplementary Figure 5. **FACS sorting of Yae62β-tg T cell subsets for TCR sequencing.** Plots show representative staining and gating used to sort the indicated T cell subsets from the **a** thymus, **b** spleen, or **c** small intestine, of wild-type or *Zap70*$^{mrd/mrt}$ Yae62β-tg mice. Analysis of the TCR sequencing data is shown in Supplementary Figure 6.

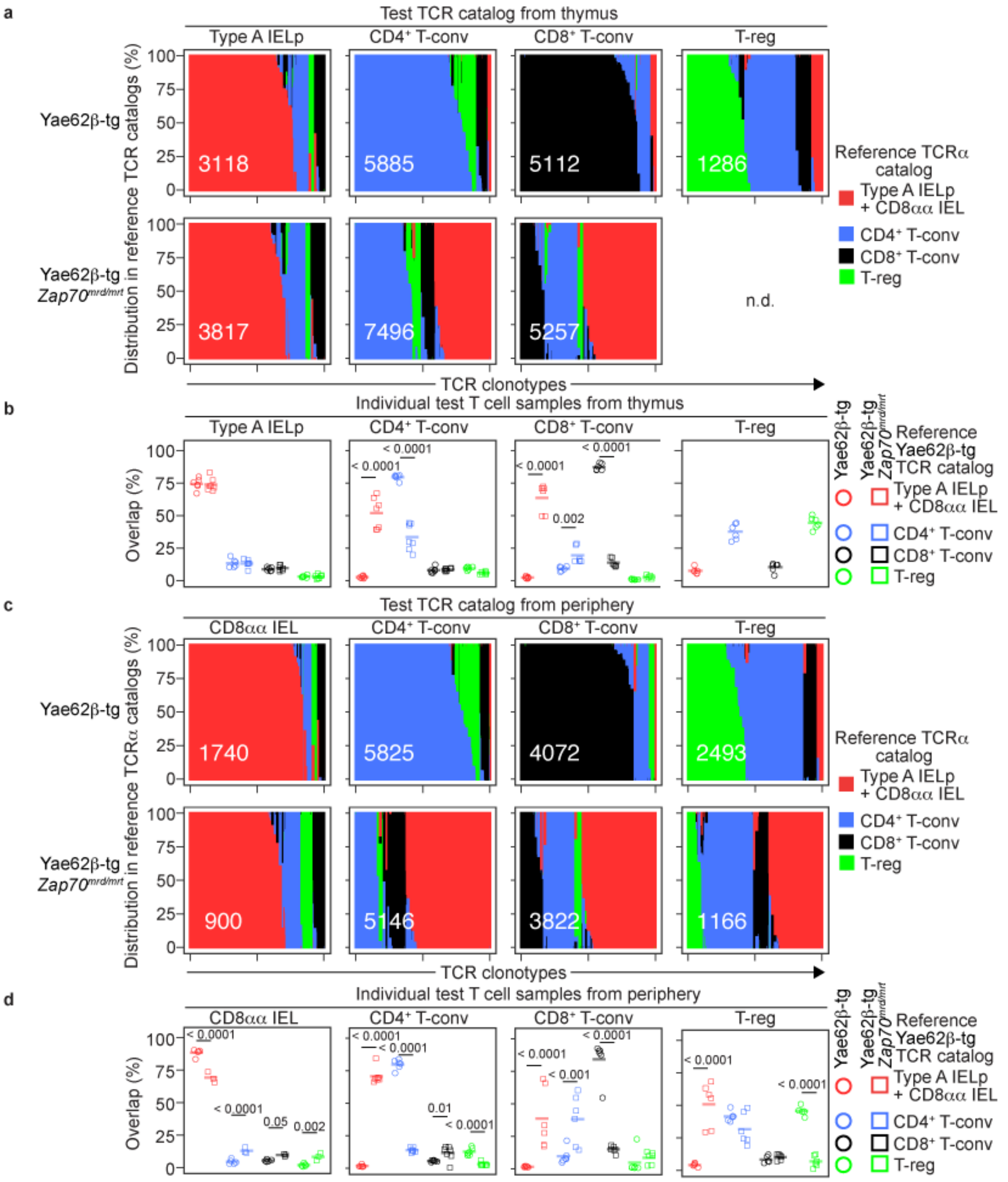

Supplementary Figure 6. **Self-reactive T cells escape thymic tolerance in *Zap70$^{mrd/mrt}$* mice.** TCRα sequences expressed in Yae62β-tg mice[30] were used to derive, for each TCR clonotype, a "Distribution in reference TCR catalogs" (y-axis, see Methods) across 4 lineages: (i) Type A IELp/CD8αα IEL; (ii) CD4$^+$ T-conv (blue); (iii) CD8$^+$ T-conv (black) and (iv) T-reg (green). TCR clonotypes expressed in **a**-**b** thymus or **c**-**d** small intestine or spleen (periphery) of male Yae62β-tg (n = 6) or Yae62β-tg *Zap70$^{mrd/mrt}$* (n = 7) mice (test samples) were aggregated to form test TCR catalogs. In **a** and **c,** each clonotype present in test and reference TCR catalogs is a column coloured based on its "Distribution in reference TCR catalogs", with "overlapping" clonotypes enumerated in white. In **b** and **d**, the "Overlap (%)" was determined by analysing each test sample as in **a** and **c**, then expressing the area of overlap with each reference TCR catalog as a percentage. In **b** and **d**, Yae62β-tg and Yae62β-tg *Zap70$^{mrd/mrt}$* test samples are represented by circles and squares, respectively, and horizontal lines show the group means. P values were determined using 2-way ANOVA, with repeated measures by sample, and Sidak's multiple comparisons test; n.d., not done. Source data are provided as a Source Data file.

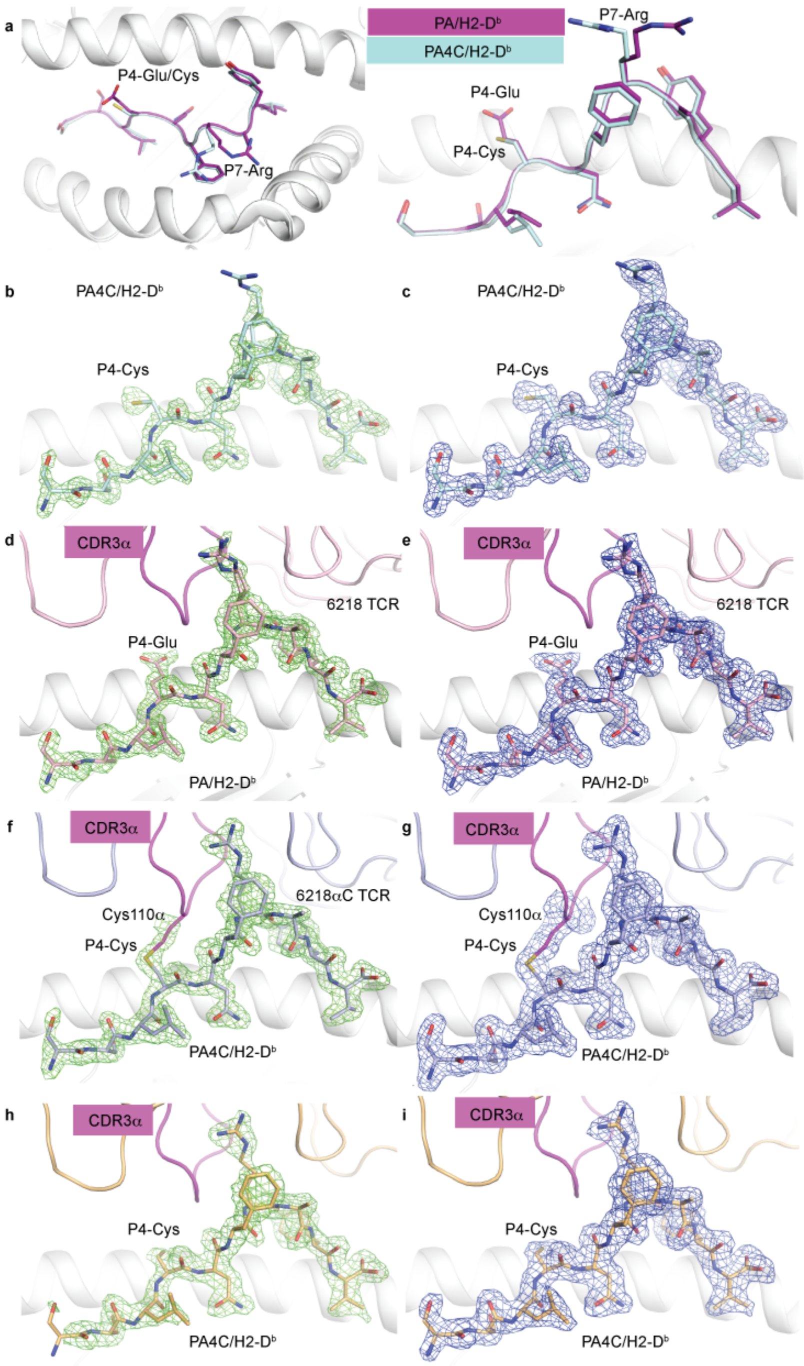

Supplementary Figure 7. **PA4C/H2-D^b structure and electron density maps of the crystal structures. a** Top (left) and side (right) views of the superposition of the PA/H2-D^b (peptide in purple) and PA4C/H2-D^b (peptide in pale blue) structures with H2-D^b in white cartoon. **b-i** Electron density maps around the PA or PA4C peptides (shown in stick) bound by H2-D^b (white on all panels), with in blue the 2Fo-Fc maps contoured at 1 σ and in green the Fo-Fc maps contoured at 3 σ. **b-c** PA4C (pale blue) in H2-D^b without TCR. **d-e** PA peptide (pink) interacting with the 6218 TCR (pink). **f-g** PA4C peptide (purple) interacting with the 6218αC TCR (purple) with the Cys110α represented as stick. **h-i** PA4C peptide (gold) interacting with the 6218 TCR (gold). The TCR CDR3α is in magenta.

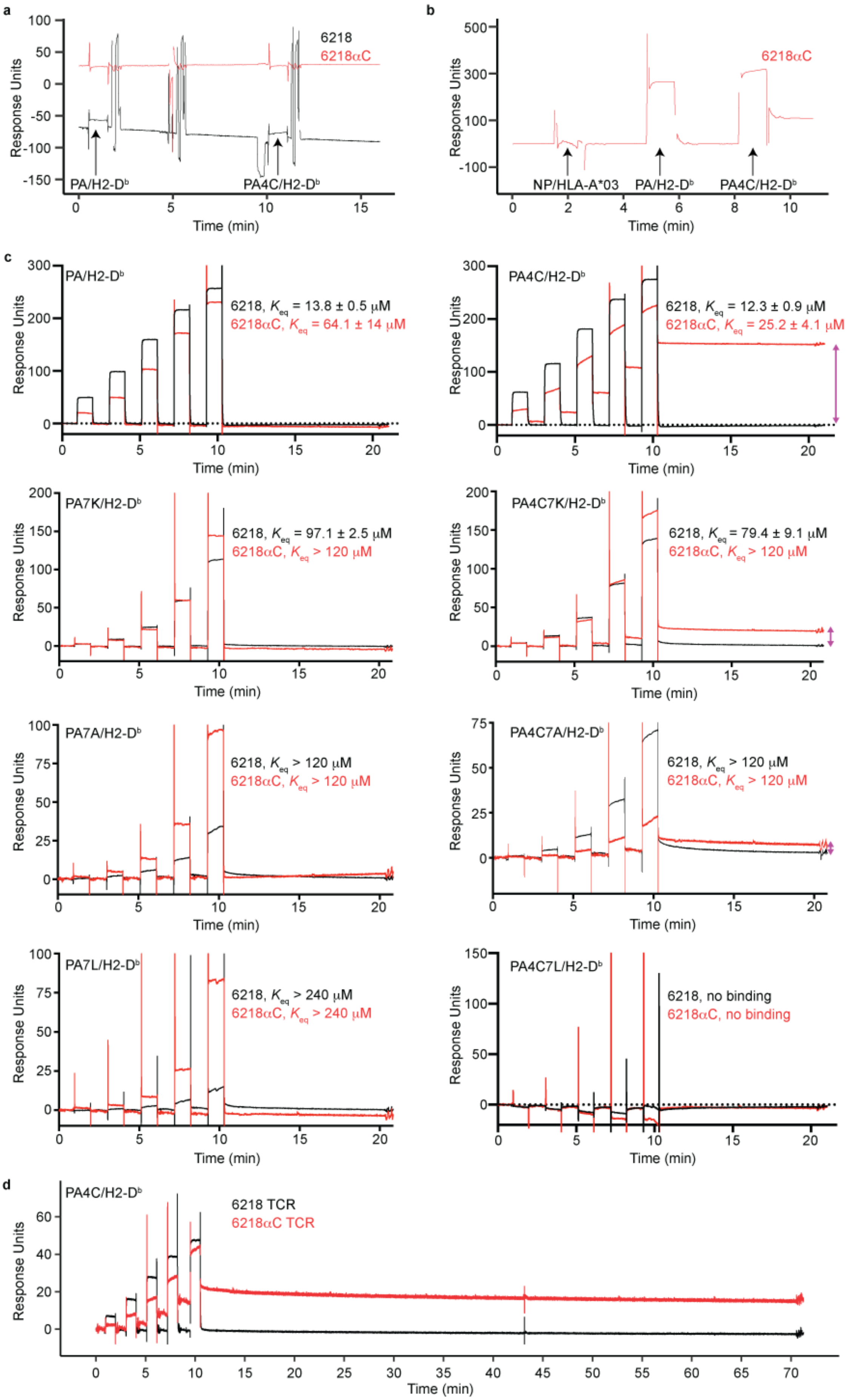

a
6218
6218αC
PA/H2-D^b
PA4C/H2-D^b
Response Units
Time (min)
b
6218αC
NP/HLA-A*03
PA/H2-D^b
PA4C/H2-D^b
Response Units
Time (min)
c
PA/H2-D^b
6218, K_{eq} = 13.8 ± 0.5 μM
6218αC, K_{eq} = 64.1 ± 14 μM
PA4C/H2-D^b
6218, K_{eq} = 12.3 ± 0.9 μM
6218αC, K_{eq} = 25.2 ± 4.1 μM
PA7K/H2-D^b
6218, K_{eq} = 97.1 ± 2.5 μM
6218αC, K_{eq} > 120 μM
PA4C7K/H2-D^b
6218, K_{eq} = 79.4 ± 9.1 μM
6218αC, K_{eq} > 120 μM
PA7A/H2-D^b
6218, K_{eq} > 120 μM
6218αC, K_{eq} > 120 μM
PA4C7A/H2-D^b
6218, K_{eq} > 120 μM
6218αC, K_{eq} > 120 μM
PA7L/H2-D^b
6218, K_{eq} > 240 μM
6218αC, K_{eq} > 240 μM
PA4C7L/H2-D^b
6218, no binding
6218αC, no binding
d
PA4C/H2-D^b
6218 TCR
6218αC TCR
Response Units
Time (min)

Supplementary Figure 8. **SPR determination of TCR-pMHC equilibrium constants (K_{eq} values) and extended persistence of 6218αC TCR-PA4C/H2-D^b complexes. a** Sensorgrams of 6218 and 6218αC TCRs without DTT treatment. Injections of 10 μM PA/H2-D^b and PA4C/H2-D^b were flowed over the biosensor chip. The 6218 TCR (black traces throughout) shows a binding response to both pMHC injections whereas the 6218αC TCR (red traces throughout) shows no binding in response to these pMHC injections. **b** Routine to enable and quantify pMHC binding to the immobilized 6218αC TCR. Immobilized 6218αC TCR was exposed to a 10-min injection of 1 mM DTT at 10 μL/min to remove TCR dimers, then the flow cell was equilibrated for 3 h in running buffer (no DTT) to reach a steady baseline. Sequential 1-min injections of a negative control pMHC (Influenza Virus $NP_{265-274}$/HLA-A*03) and a positive control pMHC (PA/H2-D^b) preceded the test pMHC monomer injections. Response units measured during PA/H2-D^b injection were used to account for differences in the amount of immobilized 6218αC TCR between sensor chips (see Methods). **c** SPR sensorgrams for immobilized 6218 or 6218αC TCRs exposed to the soluble pMHC monomer annotated in the upper left corner of each graph. The sensorgrams on the left show the PA peptide and its variants, while the sensorgrams on the right are for the PA4C peptide and its variants. Each panel shows the K_{eq} values derived from the sensorgams. The pink arrows show the retention of analyte after injection, consistent with S-S bond formation. **d** Persistent binding of PA4C/H2-D^b to 6218αC TCR. SPR sensorgrams of immobilized 6218 or 6218αC TCRs exposed to PA4C/H2-D^b sequentially injected at increasing concentrations followed by injection of buffer for 1 h. Source data are provided as a Source Data file.

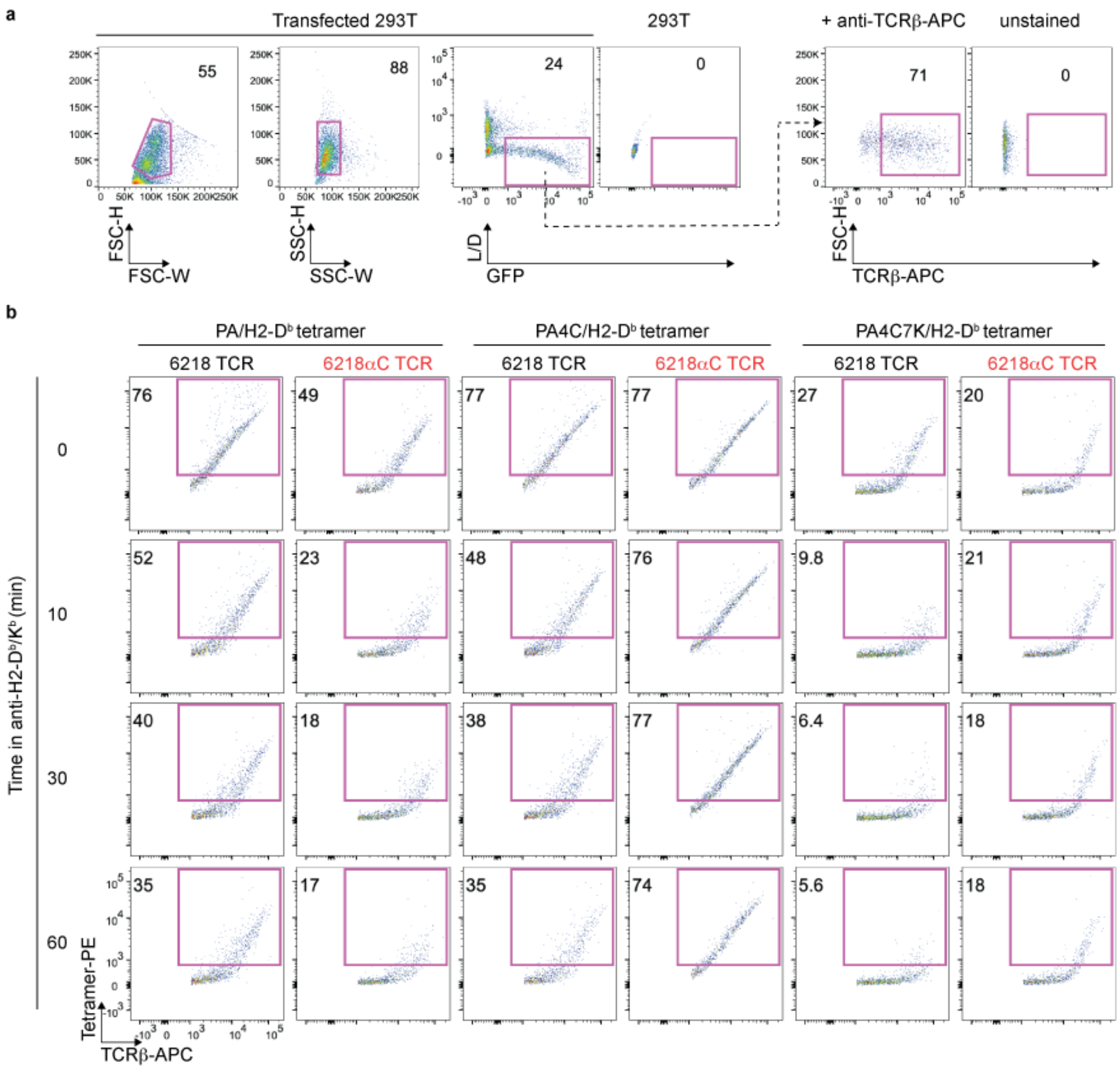

Supplementary Figure 9. **FACS gating of tetramer-binding TCR transfectants.** 293T cells transfected with plasmids encoding mouse CD3, GFP and the 6218 or 6218αC TCR were incubated for 1 h with phycoerythrin (PE)-conjugated PA/H2-D^b, PA4C/H2-D^b or PA4C7K/H2-D^b tetramers. Cells were washed and incubated for 0, 10, 30, or 60 min with anti-H2-D^b/K^b to prevent tetramer rebinding, then washed and stained with anti-TCRβ-APC and LIVE/DEAD™ Fixable Aqua Dead Cell Stain (L/D) before flow cytometric analysis. **a** FACS plots show gating for viable (L/$D^–$) GFP^+ $TCRβ^+$ cells. The plots are also representative of gating used to make Figures 4 and 7a. **b** Plots show tetramer versus TCRβ staining on the L/$D^–$ GFP^+ $TCRβ^+$ population, with numbers indicating the percentage of cells in the $tetramer^+$ gate. Data obtained in 2 experiments is graphed in Figure 7c, with source data provided as a Source Data file.

Supplementary Table 1. **Data collection and refinement statistics**

**Data Collection Statistics**	**6218 PA/H-2D[b]**	**6218 PA4C/H-2D[b]**	**6218αC PA4C/H-2D[b]**	**PA4C/H-2D[b]**
Space Group	P 2_1	P 2_1	P 2_1	P 1
Cell Dimensions (a,b,c) (Å)	54.14 72.53 107.72 β = 101.36°	54.33 72.06 107.67 β = 101.28°	57.09 74.11 114.29 β = 99.34°	46.41, 61.77, 71.68 111.33° 91.19° 91.76°
Resolution (Å)	44.07 - 1.89 (1.89 - 1.85)	44.22 - 2.09 (2.15 - 2.09)	47.41 - 1.91 (1.91 - 1.87)	46.36 - 1.76 (1.79 - 1.76)
Total number of observations	247148 (15491)	321778 (21428)	546387 (32501)	261890 (14947)
Nb of unique obs	69886 (4284)	48373 (3680)	77867 (4626)	71639 (4047)
Multiplicity	3.5 (3.6)	6.7 (5.8)	7.0 (7.0)	3.7 (3.7)
Data Completeness (%)	100.0 (100.0)	99.9 (98.9)	100.0 (100.0)	97.5 (96.3)
I/σ_I	8.7 (1.9)	11.0 (2.2)	12.9 (1.9)	9.4 (2.4)
R_{pim}^a (%)	5.6 (41.3)	4.6 (46.2)	2.9 (48.0)	5.7 (55.2)
$CC_{1/2}$ (%)	99.4 (66.7)	99.7 (66.1)	99.8 (72.4)	99.6 (63.0)
**Refinement Statistics**				
Rfactor[b](%)	18.6	19.0	20.7	18.2
Rfree[b](%)	21.4	23.6	23.7	21.8
rmsd from ideality				
Bond lengths (Å)	0.010	0.008	0.010	0.010
Bond angles (°)	1.09	1.01	1.08	1.05
Ramachandran plot (%)				
Favoured	98.2	96.4	97.5	97.7
Allowed	1.8	2.9	2.2	2.3
Disallowed	0	0.7	0.3	0
**PDB Code**	7N4K	7N5P	7N5C	7N5Q

[a]$R_{p.i.m} = \Sigma_{hkl} [1/(N-1)]^{1/2} \Sigma_i | I_{hkl,i} - \langle I_{hkl} \rangle | / \Sigma_{hkl} \langle I_{hkl} \rangle$.

[b]$R_{factor} = \Sigma_{hkl} \,||F_o| - |F_c|| / \Sigma_{hkl} |F_o|$ for all data except ≈ 5% which were used for R_{free} calculation. Values in parentheses are for the highest resolution-shell.

Supplementary Table 2. **Antibodies used in this study.**

Target	Fluorochrome	Manufacturer	Cat. No.	Clone	Application	Dilution
CD197 (CCR7)	Biotin	BioLegend	120104	4B12	FACS	400
CD197 (CCR7)	PE	BioLegend	120105	4B12	FACS	400
CD45	Vioblue	Miltenyi Biotec	130-102-430	30F11	FACS	100
CD8α	APC-fire750	BioLegend	100766	53-6.7	FACS	200
CD8β.2	PE-Cyanine7	BioLegend	140416	53-5.8	FACS	200
Helios	Pacific Blue	BioLegend	137220	22F6	FACS	200
TCRβ	BV510	BioLegend	109233	H57-597	FACS	200
CD4	AF700	BioLegend	100430	GK1.5	FACS	400
CD279 (PD-1)	BV421	BioLegend	135218	29F.1A12	FACS	200
NK1.1	PE	Miltenyi Biotec	130-102-400	PK136	FACS	200
TCRβ	APC	BioLegend	109212	H57-597	FACS	200
CD5	PerCPVio700	Miltenyi Biotec	130-103-796	53-7.3	FACS	200
CD24	PEVio770	Miltenyi Biotec	130-102-736	M1/69	FACS	800
H-2D^b/K^b	None	BD Biosciences	553575	28-8-6	Tetramer dissociation	20
CD3ε	None	BD Biosciences	553058	145-2C11	Co-culture	50
GFP	None	Abcam	ab13970	Polyclonal	Immunofluorescence histology	200
Cytokeratin 14 (K14)	None	Abcam	ab197893	EPR17336	Immunofluorescence histology	200
IgY	AF647	Abcam	ab150175	Polyclonal	Immunofluorescence histology	500
IgG	AF488	Jackson ImmunoResearch	711-545-152	Polyclonal	Immunofluorescence histology	500

Supplementary Table 3. **Oligonucleotides used for TCR sequencing.** The rightmost 2 columns indicate whether the oligonucleotide was used in the TCR sequencing experiment(s) on the fully polyclonal samples represented in Figure 3 and/or the Yae62β-tg samples represented in Supplementary Figure 6.

Target	Sequence	Polyclonal	Yae62β-tg
Trav1	TCGTCGGCAGCGTCAGATGTGTATAAGAGACAGGCACATACAGCACCTCAG	TRUE	TRUE
Trav2	TCGTCGGCAGCGTCAGATGTGTATAAGAGACAGCCACCAGGGACCACAG	FALSE	TRUE
Trav2	TCGTCGGCAGCGTCAGATGTGTATAAGAGACAGACTCTGAGCCTGCCCT	FALSE	TRUE
Trav3	TCGTCGGCAGCGTCAGATGTGTATAAGAGACAGGGCGAGCAGGTGGAG	FALSE	TRUE
Trav3	TCGTCGGCAGCGTCAGATGTGTATAAGAGACAGAACGACTCTCTCTGMACMTCACAG	TRUE	FALSE
Trav3N-2	TCGTCGGCAGCGTCAGATGTGTATAAGAGACAGGGAAAGCAGGTGGAG	FALSE	TRUE
Trav4	TCGTCGGCAGCGTCAGATGTGTATAAGAGACAGTCTGSTCTGAGATGCAATTTT	TRUE	TRUE
Trav5-1/5-4(D)	TCGTCGGCAGCGTCAGATGTGTATAAGAGACAGCTTCCYTTGGTATAAGCAAGA	TRUE	TRUE
Trav6-1/6-2	TCGTCGGCAGCGTCAGATGTGTATAAGAGACAGCAGATGCAAGGTCAAGTGAC	TRUE	TRUE
Trav6-3/6-4(D)	TCGTCGGCAGCGTCAGATGTGTATAAGAGACAGAAGGTCCACAGCTCCTTC	FALSE	TRUE
Trav6-3/6-4(D)	TCGTCGGCAGCGTCAGATGTGTATAAGAGACAGCAACTGCCAACAACAAGG	TRUE	TRUE
Trav6-6	TCGTCGGCAGCGTCAGATGTGTATAAGAGACAGAGATTCCGTGACTCAAACAG	TRUE	TRUE
Trav6(D-N)-7	TCGTCGGCAGCGTCAGATGTGTATAAGAGACAGGCCTCAAGGGACAAAGAG	TRUE	TRUE
Trav6(D)-5	TCGTCGGCAGCGTCAGATGTGTATAAGAGACAGCTTCTCTGACTGTGAACTGTTC	TRUE	TRUE
Trav7	TCGTCGGCAGCGTCAGATGTGTATAAGAGACAGAGAAGGTRCAGCAGAGCCCAGAATC	FALSE	TRUE
Trav7	TCGTCGGCAGCGTCAGATGTGTATAAGAGACAGAGAATGTGCAGCAGAGCCCAGAATC	FALSE	TRUE
Trav7	TCGTCGGCAGCGTCAGATGTGTATAAGAGACAGCATGGCCTCTCTCAACTGCAC	TRUE	FALSE
Trav8	TCGTCGGCAGCGTCAGATGTGTATAAGAGACAGTGAAYTGYAGTTACAAGAC	FALSE	TRUE
Trav8	TCGTCGGCAGCGTCAGATGTGTATAAGAGACAGAGAGCCACCCTTGACAC	TRUE	TRUE
Trav9	TCGTCGGCAGCGTCAGATGTGTATAAGAGACAGCTCKSTGSAGCTGAGATGCAA	FALSE	TRUE
Trav9	TCGTCGGCAGCGTCAGATGTGTATAAGAGACAGGGCTTTGAGGCTGAGTT	TRUE	FALSE
Trav10	TCGTCGGCAGCGTCAGATGTGTATAAGAGACAGGGAGAGAAGGTCGAGCAAC	TRUE	TRUE
Trav11	TCGTCGGCAGCGTCAGATGTGTATAAGAGACAGAAGACCCAAGTGGAGCAG	TRUE	TRUE
Trav12	TCGTCGGCAGCGTCAGATGTGTATAAGAGACAGGACCCAGAMRGAAGGCCTG	FALSE	TRUE
Trav12	TCGTCGGCAGCGTCAGATGTGTATAAGAGACAGGGTTCCACGCCACTC	TRUE	FALSE
Trav12-4	TCGTCGGCAGCGTCAGATGTGTATAAGAGACAGGGGAGGAGCAATGGAGATGG	FALSE	TRUE
Trav13	TCGTCGGCAGCGTCAGATGTGTATAAGAGACAGTCCTTGGTTCTGCAGG	TRUE	TRUE
Trav14	TCGTCGGCAGCGTCAGATGTGTATAAGAGACAGGCAGCAGGTGAGACAAAG	TRUE	TRUE
Trav15	TCGTCGGCAGCGTCAGATGTGTATAAGAGACAGCTGSAYTGTTCATATRAGACAAGT	TRUE	TRUE
Trav16	TCGTCGGCAGCGTCAGATGTGTATAAGAGACAGGTACAAGCAAACAGCAAGTG	FALSE	TRUE
Trav16	TCGTCGGCAGCGTCAGATGTGTATAAGAGACAGCTAAGCCTGTTGGGAGCAGC	FALSE	TRUE
Trav16	TCGTCGGCAGCGTCAGATGTGTATAAGAGACAGATTATTCTCTGAACTTTCAGAAGC	TRUE	FALSE
Trav17	TCGTCGGCAGCGTCAGATGTGTATAAGAGACAGCAGTCCGTGGACCAGC	TRUE	TRUE
Trav18	TCGTCGGCAGCGTCAGATGTGTATAAGAGACAGCAAGATTTCACTGCACG	FALSE	TRUE
Trav18	TCGTCGGCAGCGTCAGATGTGTATAAGAGACAGTACTGGTACCGACAGGTC	TRUE	TRUE
Trav19	TCGTCGGCAGCGTCAGATGTGTATAAGAGACAGCAAGTTAAACAAAGCTCTCCATC	TRUE	TRUE
Trav21	TCGTCGGCAGCGTCAGATGTGTATAAGAGACAGGTGCACTTGCCTTGTAGC	TRUE	TRUE
Trac	GTCTCGTGGGCTCGGAGATGTGTATAAGAGACAGNNNNNNNNNNCAGGTTCTGGGTTCTGG	TRUE	TRUE
Trbv1	TCGTCGGCAGCGTCAGATGTGTATAAGAGACAGGTATCCCTGGATGAGCTG	TRUE	FALSE
Trbv2	TCGTCGGCAGCGTCAGATGTGTATAAGAGACAGGGACAATCAGACTGCCTC	TRUE	FALSE
Trbv3	TCGTCGGCAGCGTCAGATGTGTATAAGAGACAGGATATGGGGCAGATGGTG	TRUE	FALSE
Trbv4	TCGTCGGCAGCGTCAGATGTGTATAAGAGACAGCAGGTGGGAAATGAAGTG	TRUE	FALSE
Trbv5	TCGTCGGCAGCGTCAGATGTGTATAAGAGACAGGCCAGAGCTCATGTTTCTC	TRUE	FALSE
Trbv12	TCGTCGGCAGCGTCAGATGTGTATAAGAGACAGCCAGCAGATTCTCAGTCC	TRUE	FALSE
Trbv13	TCGTCGGCAGCGTCAGATGTGTATAAGAGACAGGTACTGGTATCGGCAGGAC	TRUE	FALSE
Trbv14	TCGTCGGCAGCGTCAGATGTGTATAAGAGACAGGGTATCAGCAGCCCAGAG	TRUE	FALSE
Trbv15	TCGTCGGCAGCGTCAGATGTGTATAAGAGACAGGTGTGAGCCAGTTTCAGG	TRUE	FALSE
Trbv16	TCGTCGGCAGCGTCAGATGTGTATAAGAGACAGGAAGCAACTCTGTGGTGTG	TRUE	FALSE
Trbv17	TCGTCGGCAGCGTCAGATGTGTATAAGAGACAGGAACAGGGAAGCTGACAC	TRUE	FALSE
Trbv19	TCGTCGGCAGCGTCAGATGTGTATAAGAGACAGGGTACCGACAGGATTCAG	TRUE	FALSE
Trbv20	TCGTCGGCAGCGTCAGATGTGTATAAGAGACAGGCTTGGTATCGTCAATCG	TRUE	FALSE
Trbv23	TCGTCGGCAGCGTCAGATGTGTATAAGAGACAGGCCAGGAAGCAGAGATG	TRUE	FALSE
Trbv24	TCGTCGGCAGCGTCAGATGTGTATAAGAGACAGGCACACTGCCTTTTACTGG	TRUE	FALSE
Trbv26	TCGTCGGCAGCGTCAGATGTGTATAAGAGACAGGAGGTGTATCCCTGAAAAGG	TRUE	FALSE
Trbv29	TCGTCGGCAGCGTCAGATGTGTATAAGAGACAGGTACTGGTATCGACAAGACCC	TRUE	FALSE
Trbv30	TCGTCGGCAGCGTCAGATGTGTATAAGAGACAGGGACATCTGTCAAAGTGGC	TRUE	FALSE
Trbv31	TCGTCGGCAGCGTCAGATGTGTATAAGAGACAGCTGTTGGCCAGGTAGAGTC	TRUE	FALSE
Trbc	GTCTCGTGGGCTCGGAGATGTGTATAAGAGACAGNNNNNNNNNNGGTGGAGTCACATTTCTCAGATCC	TRUE	FALSE
