# Supplementary material for: Covalent TCR-peptide-MHC interactions induce T cell activation and redirect T cell fate in the thymus
Source: Nat Commun. 2022 Aug 23;13:4951. doi: 10.1038/s41467-022-32692-4 (PMC9399087; doi:10.1038/s41467-022-32692-4)
Supplement: Supplementary file 3 — Reporting Summary [file 41467_2022_32692_MOESM3_ESM.pdf]

nature portfolio

Corresponding author(s): Stephen R. Daley

Last updated by author(s): Aug 8, 2022

Reporting Summary

Nature Portfolio wishes to improve the reproducibility of the work that we publish. This form provides structure for consistency and transparency in reporting. For further information on Nature Portfolio policies, see our Editorial Policies and the Editorial Policy Checklist.

Statistics

For all statistical analyses, confirm that the following items are present in the figure legend, table legend, main text, or Methods section.

n/a	Confirmed	
[ ]	[x]	The exact sample size (*n*) for each experimental group/condition, given as a discrete number and unit of measurement
[ ]	[x]	A statement on whether measurements were taken from distinct samples or whether the same sample was measured repeatedly
[ ]	[x]	The statistical test(s) used AND whether they are one- or two-sided<br>*Only common tests should be described solely by name; describe more complex techniques in the Methods section.*
[x]	[ ]	A description of all covariates tested
[ ]	[x]	A description of any assumptions or corrections, such as tests of normality and adjustment for multiple comparisons
[ ]	[x]	A full description of the statistical parameters including central tendency (e.g. means) or other basic estimates (e.g. regression coefficient) AND variation (e.g. standard deviation) or associated estimates of uncertainty (e.g. confidence intervals)
[ ]	[x]	For null hypothesis testing, the test statistic (e.g. *F*, *t*, *r*) with confidence intervals, effect sizes, degrees of freedom and *P* value noted<br>*Give P values as exact values whenever suitable.*
[x]	[ ]	For Bayesian analysis, information on the choice of priors and Markov chain Monte Carlo settings
[x]	[ ]	For hierarchical and complex designs, identification of the appropriate level for tests and full reporting of outcomes
[x]	[ ]	Estimates of effect sizes (e.g. Cohen's *d*, Pearson's *r*), indicating how they were calculated

Our web collection on statistics for biologists contains articles on many of the points above.

Software and code

Policy information about availability of computer code

Data collection	X-ray crystallography data were collected using AS-GUI, a graphical user interface collection system developed at the Australian Synchrotron, Australian Nuclear Science and Technology Organisation.

Data analysis	TCR sequences were aligned to mouse TCR genes using molecular identifier groups-based error correction (MIGEC) software (version 1.2.6). Code, processed TCR sequence data, and examples of expected output have been deposited in GitHub and are available at https://zenodo.org/badge/latestdoi/514173665. The 'tidyverse' (version 1.3.1) and 'stringr' (version 1.4.0) packages were used to perform TCR sequence analyses and produce graphs in RStudio (version 2022.02.0 Build 443).<br>X-ray crystallography data were processed using XDS (BUILT=20161205). Data were scaled and reduced using the Pointless and Aimless program from the CCP4 suite (version 7.0.077). Structures were determined by molecular replacement using the PHASER program (version 2.8.3) from the CCP4 suite (1994) (version 7.0.077) with a model of H2-Db without the peptide derived from PDB ID: 3PQY. Manual model building was conducted using COOT (version 0.8.9.2) followed by refinement with BUSTER (version 2.10.3). Molecular graphics representations were created using PyMOL (version 2.5.0a0).<br>Surface plasmon resonance data were exported using BIAevaluation 3.0 Software.<br>Flow cytometry data were acquired using BD FACSDiva Software (version 1.2.0.142) and analysed using FlowJo software (version 10.8.1).<br>Fiji (version 2.3.0/1.53f) was used to quantify GFP+ cells, and cortical and medullary areas, in immunofluorescence histology sections.<br>The software used to make immunofluorescence images for Figure 2 was cellSens Dimension version 4.1 (Olympus Corporation, Tokyo, Japan).<br>Statistical analyses were performed using GraphPad Prism (Version 9.3.1 (350)). Figures were made using Adobe Illustrator 2021 (version 25.4.5) or Microsoft PowerPoint (version 16.63.1).

For manuscripts utilizing custom algorithms or software that are central to the research but not yet described in published literature, software must be made available to editors and reviewers. We strongly encourage code deposition in a community repository (e.g. GitHub). See the Nature Portfolio guidelines for submitting code & software for further information.

Data

Policy information about availability of data

All manuscripts must include a data availability statement. This statement should provide the following information, where applicable:

- Accession codes, unique identifiers, or web links for publicly available datasets
- A description of any restrictions on data availability
- For clinical datasets or third party data, please ensure that the statement adheres to our policy

The TCR sequencing data generated in this study have been deposited in the NCBI Short Read Archive under BioProject ID "PRJNA733833 [https://www.ncbi.nlm.nih.gov/bioproject/?term=PRJNA733833]" for Fig. 3 and "PRJNA734126 [https://www.ncbi.nlm.nih.gov/bioproject/?term=PRJNA734126]"for Supplementary Fig. 6.
The crystal structure coordinates have been deposited in the Protein Data Bank (PDB) under the following accession codes: "7N5Q [http://doi.org/10.2210/pdb7n5q/pdb]" for PA4C/H2-Db, "7N4K [http://doi.org/10.2210/pdb7n4k/pdb]" for 6218-PA/H2-Db, "7N5P [http://doi.org/10.2210/pdb7n5p/pdb]" for 6218-PA4C/H2-Db, and "7N5C [http://doi.org/10.2210/pdb7n5c/pdb]" for 6218aC-PA4C/H2-Db.
All data are included in the Supplemental Information or available from the authors upon reasonable requests, as are unique reagents used in this Article. The raw numbers for charts and graphs are available in the Source Data file whenever possible.

Human research participants

Policy information about studies involving human research participants and Sex and Gender in Research.

Reporting on sex and gender	This study did not involve human research participants.
Population characteristics	Not applicable.
Recruitment	Not applicable.
Ethics oversight	Not applicable.

Note that full information on the approval of the study protocol must also be provided in the manuscript.

Field-specific reporting

Please select the one below that is the best fit for your research. If you are not sure, read the appropriate sections before making your selection.

[x] Life sciences [ ] Behavioural & social sciences [ ] Ecological, evolutionary & environmental sciences

For a reference copy of the document with all sections, see nature.com/documents/nr-reporting-summary-flat.pdf

Life sciences study design

All studies must disclose on these points even when the disclosure is negative.

Sample size	For TCR sequencing and TCR-retrogenic studies, sample size was determined by the availability of mice and human resources to perform experiments. Surface plasmon resonance, tetramer staining of TCR transfectants, and co-culture experiments typically included 2 technical replicates for each condition. Except for tetramer staining of 6218bC transfectants, which was done once, each experiment was repeated once to confirm the results were reproducible. Additional repeats were sometimes performed to test the effects of new reagents or

conditions on the results. The reported sample sizes were deemed sufficient based on the small amount of inter-experiment variation and the significance of statistical differences in the results between conditions. For X-ray crystallography, the reported data was deemed sufficient based on the refinement statistics, as described in Supplementary Table 1.

Data exclusions

Fig 1. One mouse from the 6218 group and one mouse from the 6218aC group died on day 8 and day 33 after transplantation, respectively; these mice were excluded.
Fig 2. In the immunofluorescence histology experiment, in thymic sections from 4 TCR-retrogenic mice (3 in the 6218 group and 1 in the 6218aC group), the number of GFP+ cells per mm^2 was within 2 standard deviations of the mean of thymic sections from negative control B6 mice (n = 6). These samples were excluded based on this predetermined criterion for inclusion.
Fig 3a. Two recipient mice from each group died after BM transplantation (on day 11 and day 40 in the 6218 group; on day 11 and day 12 in the 6218aC group; and on day 11 and day 32 in the 6218bC group); these mice were excluded.
Supp. Fig 2. Two recipient mice in the 6218aC group died on day 8 and day 10 after BM transplantation; these mice were excluded.

Replication

Except for tetramer staining of 6218bC transfectants, which was done once, producing a complete absence of tetramer binding, each experiment was done at least twice. In all experiments in which the positive and negative controls produced expected results, attempts at replication were successful.

Randomization

For TCR sequencing studies, mice were allocated into experimental groups based on the results of genotyping assays. For TCR-retrogenic studies, cages of mice were allocated to a single experimental group until groups reached the maximum size that was manageable with the mouse and human resources available. Randomization is not relevant to X-ray crystallography, surface plasmon resonance, tetramer staining of TCR transfectants, and co-culture experiments because samples are allocated to experimental groups based on differences that are intrinsic to the samples.

Blinding

Researchers were blinded to the experimental groups of the thymus immunofluorescence histology samples during the counting of GFP+ cells and determination of cortical and medullary areas. Otherwise, researchers were not blinded because it was necessary to allocate samples to experimental groups based on differences intrinsic to the samples.

Reporting for specific materials, systems and methods

We require information from authors about some types of materials, experimental systems and methods used in many studies. Here, indicate whether each material, system or method listed is relevant to your study. If you are not sure if a list item applies to your research, read the appropriate section before selecting a response.

Materials & experimental systems

n/a	Involved in the study	
☐	☒	Antibodies
☐	☒	Eukaryotic cell lines
☒	☐	Palaeontology and archaeology
☐	☒	Animals and other organisms
☒	☐	Clinical data
☒	☐	Dual use research of concern

Methods

n/a	Involved in the study	
☒	☐	ChIP-seq
☐	☒	Flow cytometry
☒	☐	MRI-based neuroimaging

Antibodies

Antibodies used

Anti-CD197 (CCR7)-Biotin,4B12,Biolegend,120104,1:400
Anti-CD197 (CCR7)-PE,4B12,Biolegend,120105,1:400
Anti-CD45-Vioblue,30F11,Miltenyi,130-102-430,1:100
Anti-CD8a-APC-fire750,53-6.7,Biolegend,100766,1:200
Anti-CD8b.2-PE-Cyanine7,53-5.8,Biolegend,140416,1:200
Anti-Helios-Pacific Blue,22F6,Biolegend,137220,1:200
Anti-TCRβ-BV510,H57-597,Biolegend,109233,1:200
Anti-CD4-AF700,GK1.5,Biolegend,100430,1:400
Anti-CD279 (PD-1)-BV421,29F.1A12,Biolegend,135218,1:200
Anti-NK1.1-PE,PK136,Miltenyi,130-102-400,1:200
Anti-TCRb-APC,H57-597,Biolegend,109212,1:200
Anti-CD5-PerCPVio700,53-7.3,Miltenyi,130-103-796,1:200
Anti-CD24-PEVio770,M1/69,Miltenyi,130-102-736,1:800
Anti-H-2Db/Kb-None,28/8/2006,BD Biosciences,553575,1:20
Anti-CD3e,145-2C11,BD Biosciences,553058,1:50
Anti-GFP,Polyclonal,Abcam,ab13970,1:200
Anti-Cytokeratin 14 (K14),EPR17336,Abcam,ab197893,1:200
Anti-IgY-AF647,Polyclonal,Abcam,ab150175,1:500
Anti-IgG-AF488,Polyclonal,Jackson ImmunoResearch,711-545-152,1:500

Validation

All monoclonal antibodies used are commercially available and were validated with mouse cells for flow cytometry application by the manufacturers. A validation statement for each antibody is available on the manufacturer's website.

BD Bioscience (https://www.bdbiosciences.com)
BioLegend (https://www.biolegend.com)
Miltenyi Biotec (https://www.miltenyibiotec.com/AU-en/products/macs-antibodies/antibodies-for-flow-cytometry.html)
The Chicken polyclonal anti-GFP "does cross-react with the many fluorescent proteins that are derived from the jellyfish Aequorea victoria. These are all proteins that differ from the original GFP by just a few point mutations (EGFP, YFP, mVenus, CFP, BFP etc.)" (https://www.abcam.com/gfp-antibody-ab13970.html).
The specificity of the rabbit monoclonal antibody [EPR17336] to Cytokeratin 14 is "knockout validated" (https://www.abcam.com/cytokeratin-14-antibody-epr17336-ab197893.html).
"By immunoelectrophoresis and ELISA", the Goat Anti-Chicken IgY H&L (Alexa Fluor® 647) preadsorbed antibody "reacts specifically with chicken IgG and with light chains common to other chicken immunoglobulins. No antibody was detected against non-immunoglobulin serum proteins. Reduced cross-reactivity to bovine, goat, horse, human, mouse, pig, rabbit and rat IgG was detected. This antibody may cross react with IgG from other species" (https://www.abcam.com/goat-chicken-igy-hl-alexa-fluor-647-preadsorbed-ab150175.html).
"Based on immunoelectrophoresis and/or ELISA", the Alexa Fluor® 488 AffiniPure Donkey Anti-Rabbit IgG (H+L) "antibody reacts with whole molecule rabbit IgG. It also reacts with the light chains of other rabbit immunoglobulins. No antibody was detected against non-immunoglobulin serum proteins. The antibody has been tested by ELISA and/or solid-phase adsorbed to ensure minimal cross-reaction with bovine, chicken, goat, guinea pig, syrian hamster, horse, human, mouse, rat and sheep serum proteins, but it may cross-react with immunoglobulins from other species" (https://www.jacksonimmuno.com/catalog/products/711-545-152).

Eukaryotic cell lines

Policy information about cell lines and Sex and Gender in Research

Cell line source(s)	293T, American Type Culture Collection, Manassas, VA, Cat. no. CRL-3216<br>DC2.4, Dr Kenneth Rock, University of Massachusetts, Worcester, MA<br>GP+E86, Dr Dario Vignali, University of Pittsburgh, Pittsburgh, PA<br>5KC-73.8.20, Dr Philippa Marrack, National Jewish Health, Denver, CO
Authentication	None of the cell lines used were authenticated.
Mycoplasma contamination	No cell lines were tested for Mycoplasma contamination.
Commonly misidentified lines<br>(See ICLAC register)	All cell lines used in this study are not listed in the ICLAC register of commonly misidentified lines.

Animals and other research organisms

Policy information about studies involving animals; ARRIVE guidelines recommended for reporting animal research, and Sex and Gender in Research

Laboratory animals	B6.Rag1 KO mice were purchased from Bioservices Kew, Walter and Eliza Hall Institute of Medical Research (Melbourne, Australia). C57BL/6 (B6), Tcra KO, B2m KO, H2-Aa KO, Zap70mrd , Zap70mrt , BCL2-tg (Tg(Vav-BCL2)1Jad), Yae62β-tg , Foxp3GFP, all on the B6 genetic background, male and female, were bred (intercrossed in some cases) and housed in Specific-Pathogen-Free environments at 18-24 °C and 40-70 % humidity with a lighting cycle of 7 a.m. to 7 p.m. light (below 350 lux) and 7 p.m. to 7 a.m. darkness, at the Australian Phenomics Facility, Canberra, or at Monash University, Melbourne. Mice bearing Zap70mrt (strain ID 158) or Zap70mrd (strain ID 159) alleles may be rederived from cryopreserved sperm stored at the Australian Phenomics Facility.
Wild animals	The study did not involve wild animals.
Reporting on sex	This study reports data obtained from 57 male and 36 female mice. The sex of each mouse is shown in the Source Data file. Experiments used mice of a single sex when sufficient mice of one sex were available. Otherwise, mice of both sexes were used. The findings apply to mice of both sexes.
Field-collected samples	The study did not involve samples collected from the field.
Ethics oversight	The Animal Experimentation Ethics Committee of the Australian National University (A2014/62 and A2018/06) and the Monash University Animal Experimentation Ethics Committee (MARP/2015/64) had oversight of the project.

Note that full information on the approval of the study protocol must also be provided in the manuscript.

Flow Cytometry

Plots

Confirm that:

- [x] The axis labels state the marker and fluorochrome used (e.g. CD4-FITC).
- [x] The axis scales are clearly visible. Include numbers along axes only for bottom left plot of group (a 'group' is an analysis of identical markers).
- [x] All plots are contour plots with outliers or pseudocolor plots.
- [x] A numerical value for number of cells or percentage (with statistics) is provided.

Methodology

Sample preparation	Whole thymus and spleen suspensions were prepared by pushing organs through a 70-micron sieve in sort buffer (PBS containing 2% v/v heat-inactivated fetal calf serum and 2mM EDTA). Small intestine was first cut longitudinally and then into pieces ~ 0.5 cm long while being kept moist with washing medium (WM, DMEM containing 2.5% v/v heat-inactivated bovine serum and 10mM HEPES) and placed in a 50 mL tube containing ~15 mL ice-cold WM. Intestinal contents were removed by cycles of vortexing for 5 s, then removing the supernatant by using a strainer to retain intestinal tissue and resuspending in 15 mL WM, until supernatant was clear. Tissue pieces were then incubated for 15 minutes at 37°C with gentle rotation in dissociation buffer (calcium- and magnesium-free PBS containing 5% v/v heat-inactivated bovine serum plus 2mM EDTA). After vortexing for 15 s, tissue pieces were removed using a strainer and discarded, while the supernatant was passed through a 70 μm sieve, pelleted by centrifugation, resuspended in 5 mL of 40% Percoll and overlaid onto 5 mL 80% Percoll in a 15 mL tube. After centrifugation for 20 min at 900 g at 20°C, material at the interface was collected, transferred to a fresh 15 mL tube containing 10mL of sort buffer, pelleted and incubated with antibodies. Up to 4 million cells were incubated in 50 uL FACS buffer (PBS containing 2% v/v heat-inactivated bovine serum, optionally including 0.01% m/v sodium azide if not cell sorting) for 30 min at 4°C containing assortments of fluorescently labelled antibodies followed by washing and, typically, incubation in viability dye. Samples were then washed in FACS buffer and data were acquired, or the cells were fixed and permeabilised using the Foxp3 / Transcription Factor Staining Buffer Set (Thermo Fisher Scientific, Waltham, MA, Cat. no.00-5523-00), then incubated with anti-Helios-Pacific Blue (BioLegend, Cat. no. 137220). Samples were then processed using the Click-iT® EdU Flow Cytometry Assay Kit (Thermo Fisher Scientific, Cat. no. C10420) following the manufacturer's instructions except that Click-iT® EdU buffer additive (Component G) was used at one-fifth of the concentration recommended. Samples were then washed in FACS buffer and incubated with PE-Vio770-conjugated anti-CD24 (Miltenyi, Cat. no. 130-102-736) and PE-streptavidin (BioLegend, Cat. no. 405204). After washing in FACS buffer, data was acquired on flow cytometers.
Instrument	Acquisition: BD FACS Fortessa or FACS LSR II.<br>Sorting: BD Influx Cell Sorter
Software	Collection: BD FACSDiva Software (version 1.2.0.142).<br>Analysis: FlowJo software (version 10.8.1).
Cell population abundance	Aliquots of sorted T cells were reanalysed with the instrument used for sorting and 90-98% of cells were found to have the expected phenotype.
Gating strategy	Events were initially gated by size based on FSC(H)/FSC(W) and SSC(H)/SSC(W) to exclude cell doublets. Live cells were then gated based on viability dye before analysis for cellular markers.

- [x] Tick this box to confirm that a figure exemplifying the gating strategy is provided in the Supplementary Information.
